# Discrete Element Method Simulations of the Inter-Particle Contact Parameters for the Mono-Sized Iron Ore Particles

**DOI:** 10.3390/ma10050520

**Published:** 2017-05-11

**Authors:** Tongqing Li, Yuxing Peng, Zhencai Zhu, Shengyong Zou, Zixin Yin

**Affiliations:** 1 School of Mechatronic Engineering, China University of Mining and Technology, Xuzhou 221116, China; litongqing@cumt.edu.cn (T.L.); zhuzhencai@cumt.edu.cn (Z.Z.); yinzixin@cumt.edu.cn (Z.Y.); 2 Jiangsu Key Laboratory of Mine Mechanical and Electrical Equipment, China University of Mining and Technology, Xuzhou 221116, China; 3 State Key Laboratory of Mining Heavy Equipment, CITIC Heavy Industries Co., Ltd., Luoyang 471000, China; zoushengyong@hotmail.com

**Keywords:** angle of repose, contact parameters, iron ore particles, DEM simulation, mills

## Abstract

Aiming at predicting what happens in reality inside mills, the contact parameters of iron ore particles for discrete element method (DEM) simulations should be determined accurately. To allow the irregular shape to be accurately determined, the sphere clump method was employed in modelling the particle shape. The inter-particle contact parameters were systematically altered whilst the contact parameters between the particle and wall were arbitrarily assumed, in order to purely assess its impact on the angle of repose for the mono-sized iron ore particles. Results show that varying the restitution coefficient over the range considered does not lead to any obvious difference in the angle of repose, but the angle of repose has strong sensitivity to the rolling/static friction coefficient. The impacts of the rolling/static friction coefficient on the angle of repose are interrelated, and increasing the inter-particle rolling/static friction coefficient can evidently increase the angle of repose. However, the impact of the static friction coefficient is more profound than that of the rolling friction coefficient. Finally, a predictive equation is established and a very close agreement between the predicted and simulated angle of repose is attained. This predictive equation can enormously shorten the inter-particle contact parameters calibration time that can help in the implementation of DEM simulations.

## 1. Introduction

With the rapid development of computing power and advanced contact algorithm, the discrete element method (DEM) has been extensively applied as a leading tool to describe diverse issues in granular processes, including many industrial applications, such as mining, chemical, cement, and agricultural industries, particularly tumbling mills [1,2,3,4,5,6,7,8,9,10,11,12]. DEM simulations hitherto have been demonstrated their extremely desirability to predict what happens in reality, as well as the quantitatively accurate information representation inside the mills, while the accuracy of outcomes for DEM simulations depends highly on the input parameters [13,14,15,16,17,18], including parameters such as contact parameters (restitution coefficient, static friction coefficient, and rolling friction coefficient), mechanical properties (shear modulus, Poisson’s ratio, and density), and particle shape.

Researchers studied that the DEM simulation of mill behavior was only a weak variation with the mechanical properties [13,14,15,16,19,20,21]. To dramatically accelerate the computational efficiency of DEM simulations, the reduction in shear modulus was commonly employed without changing the charge behavior [15,22,23]. In the last few decades the determination of contact parameters of charge were mainly classified into two approaches [14]. The first approach was to determine the contact parameters separately using the experimental setups [24,25,26], whereas the restrictions on particle size and shape were difficult to be measured, especially for the inter-particle contact parameters at a small scale [14,26]. At present, the DEM parameters at the mesoscale can be truly determined on the basis of molecular dynamics simulations [27,28]. The second approach was to set a series of arbitrary input parameters in DEM simulations until the results were in very close agreement to the experimental results, namely, sand-pile calibrations [29,30,31]. The angle of repose, as the key parameter in describing the granule flow characteristic, has been widely employed in calibrating the contact parameters of granule materials. Numerous experiments and DEM simulations proposed that the angle of repose was highly sensitive to the contact parameters [13,14,16,17,18]. Generally speaking, the repeated calibrations consist of two parts, namely particle-wall contact parameters and inter-particle contact parameters. Therefore, the determination of contact parameters by means of the second approach is time-consuming and sophisticated. Currently, the contact parameters between particles and the wall were commonly determined with various experimental setups in the first place, and the repeated sand-pile test was subsequently employed in calibrating the inter-particle contact parameters, in order to reduce the number of calibrations.

Iron ores are the primary source for iron- and steel-making industries, which use tumbling mills for further comminution to obtain the required particle size distribution. The shapes of iron ore particles are highly irregular due to the impact-breakage, abrasion, and attrition. Currently, the exact simulation of irregular shapes hitherto is still one of the key issues to be solved in the DEM simulations [32,33]. To trustworthily predict what happens in reality, rather than qualitative prediction, the accuracy of DEM simulations requires special consideration with the particle shape, due to the influence of packing density on the particle flow properties. Arguably, the sensitivity of the angle of repose dependence on the particle shape varied from a low value, for smooth granules, to a high value, for irregular granules [13,25,34,35]. Therefore, modelling the contact model with irregular shapes is essential and worthwhile for DEM simulations. Many investigators employed the simple spherical shape in the majority of DEM simulations to achieve a reasonable simulation time, but the shortcomings of unrealistic simplifications and assumptions are also clear. Such simplified models are an unrealistic measure on both the physical properties and contact parameters, as well as a poor particle representation on the packing characteristics. Along with the fast development of X-ray and image processing techniques, the multi-element spheres method has been used successfully to represent the geometrical model of irregular iron ore particles [36,37,38]. To achieve an exact representation of particle shape, a large number of spheres were used to describe the information of the details, but at the expense of much greater computational effort [39]. Thus, considering the accuracy in describing the shape of iron ore particles and computational effort, the appropriate geometrical shape of irregular particles should be carefully modelled. 

To reduce the simulations, as well as the computational effort, the determination of the contact parameters should be studied quantitatively for determining which parameter has the greatest impact on the angle of repose. In this paper, the irregular shapes of iron ore particles were determined, and a quick and accurate sphere clump method was employed in modelling the irregular shape. The appropriate number of spheres was quantitatively selected on the basis of the multi-element sphere method. The inter-particle contact parameters were systematically changed whilst the particle-wall contact parameters were arbitrarily assumed to purely assess their impact on the angle of repose using the split cylinder method. A predictive equation was formed, which will provide the basic data for the DEM simulations of tumbling mills.

## 2. DEM Model

The exact representation of irregular particles for DEM simulations in an efficient manner is still a major limitation in the current state for industrial applications. The exact definition of the irregular shape element hitherto is extremely sophisticated. Therefore, developing the contact model with the non-spherical methods was phenomenally complicated compared with the multi-element sphere methods. To achieve a better computational efficiency for contact detection, the multi-element sphere method was employed in modelling the approximation of any desired shape. In this study, the non-linear model, namely the no-slip Hertz–Mindlin model, was employed to figure out the contact force between particle systems within the EDEM software package (DEM—Solutions Ltd., Edinburgh, UK) because of the accurate representation of the physical situation and less computational effort. In EDEM software any kind of irregular geometrical shape of particles can be generated as a clump composed of several touching or overlapping spheres. Therefore, the contact detection between the sphere clumps is sphere-based and, therefore, the discrete element algorithm of the sphere clumps is fully available for calculating the contact forces.

**Figure 1:**
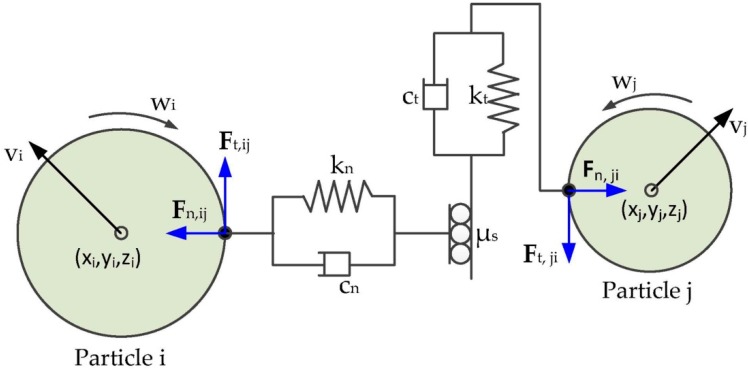
The illustration of the contact forces between particle *i* and particle *j*.

Although the simplified Hertz–Mindlin model has been addressed extensively, it is worthwhile to review the main equations again and the corresponding inputs are essential to the model. According to the Newton’s second law of motion, the translational and rotational motions of particle *j* (Figure 1) can be expressed as [31]:

(1) $$m_{j}\frac{dv_{j}}{dt}=m_{j}g+\sum_{i}(F_{n,ij}+F_{t,ij})$$

(2) $$I_{j}\frac{dw_{j}}{dt}=-\mu_{r,ij}F_{ij}R_{j}\frac{w_{j}}{\left|w_{j}\right|}+\sum_{i}R_{j}\times F_{t,ij}$$

where *m_j_*, *v_j_*, *w_j_*, *I_j_* are mass, velocity, angular velocity, and the moment of inertia of particle *j*, respectively. ***F****_n,ij_* and ***F****_t,ij_* are the normal force and total tangential force between particle *i* and *j* given as:

(3) $$F_{n,ij}=-\frac{4}{3}E^{*}\sqrt{R^{*}}\delta^{\frac{3}{2}}+\sqrt{\frac{20}{3}}\beta {\left(m^{*}E^{*}\sqrt{R^{*}\delta_{n}}\right)}^{\frac{1}{2}}v_{n,ij}$$

(4) $$F_{t,ij}=\min[\mu_{s,ij}F_{n,ij},\;8G^{*}\delta_{t}\sqrt{R^{*}\delta_{n}}+\sqrt{\frac{80}{3}}\beta {\left(m^{*}G^{*}\sqrt{R^{*}\delta_{n}}\right)}^{\frac{1}{2}}v_{t,ij}]$$

where E^*^ is equivalent Young’s modulus, 1/E^*^ = (1 − *u*_1_^2^)/*E_i_* + (1 − *u*_2_^2^)/*E_j_*, *E_i_*, *E_j_*, *u_i_*, *u_j_* are the Young’s Modulus and Poisson’s ratio of particles *i* and particle *j*, respectively; R^*^ is equivalent radius, 1/R^*^ = 1/*R_i_* + 1/*R_j_*, *R_i_* and *R_j_* are the contact radius of particles *i* and particle *j*. m^*^ is equivalent mass, 1/m^*^ = 1/*m_i_* + 1/*m_j_*, *m_i_* and *m_j_* are the mass of particles *i* and particle *j*. *δ_n_* and *δ_t_* is the normal overlap and tangential overlap, respectively; *μ_s,ij_* is the coefficient of static friction between particles *i* and particle *j*. *v_n,ij_* and *v_t,ij_* are the relative normal velocity and tangential velocity, respectively.

As mentioned above, the discrete element algorithm of the sphere clumps is fully available for calculating the contact forces. The properties of total mass and products of inertia of the clump were given as follows [36]:

(5) $$m^{ct}=\sum_{k=1}^{n}m^{\left[k\right]}$$

(6) $$x_{i}^{ct}=\frac{1}{m^{ct}}\sum_{k=1}^{n}m^{\left[k\right]}{x_{i}}^{\left[k\right]}$$

(7) $$I_{ii}=\sum_{k=1}^{n}\left\{m^{\left[k\right]}{\left({x_{j}}^{\left[k\right]}-{x_{j}}^{ct}\right)}^{2}+\frac{2}{5}m^{\left[k\right]}{\left(r^{\left[k\right]}\right)}^{2}\right\}$$

(8) $$I_{ij}=\sum_{k=1}^{n}\left\{m^{\left[k\right]}{\left({x_{j}}^{\left[k\right]}-{x_{j}}^{ct}\right)}^{2}\right\};i\neq j$$

where *m^ct^*, *x_i_^ct^* and *n* are the total mass of the clump, center of mass, and number of the balls, respectively, *m*^[*k*]^, *x*^[*k*]^, and *r*^[*k*]^ are the mass, center of mass, and radius of *k*th ball, respectively.

DEM employs the modelling of each particle as a rigid body and determining the displacement and velocity on the basis of Newton’s second law at a time step. To make a trustworthy simulation, a time steps, in the order of a millionth of a second, is required to predict what happens in reality, which leads to greater computationally expense. Researchers proposed that the appropriate time step for simulating the dense particle motion was in range of 20–80% [40,41]. The time step, defined as the time between the particle iterations, must be less than the critical Rayleigh time step Δ*T_R_*:

(9) $$\Delta T_{step}<\Delta T_{R}=\frac{\pi R_{p}}{\left(0.163v_{p}+0.8766\right)}\sqrt{\frac{\rho_{p}}{G_{p}}}$$

where *R_p_* is the particle radius, *ν_p_* the particle Poisson’s ratio, *ρ_p_* the particle density, *G_p_* the particle shear modulus. In the current study, 30% of the critical Rayleigh time step was used due to the consideration of computational effort and accuracy.

## 3. Methodology

### 3.1. Sphere Clump Method

In mineral processing, the real shape of iron ore particles is highly irregular due to the impact-breakage, abrasion, and attrition. Generally speaking, the exact definition of real particle shapes hitherto is still one of the key issues to be solved. So far there are various definitions qualitatively describing the particle shape, including parameters such as aspect ratio, sphericity, and shape factor [42]. Nevertheless, sphericity indicates how closely the particle geometry is to a perfect sphere and is employed extensively in powder technology [36]:

(10) $$\Psi =\frac{SA_{es}}{SA_{rp}}=\frac{\sqrt[{3}]{36\pi V^{2}}}{SA_{rp}}$$

where *SA_rp_* is the real surface area of the iron ore particle, mm^2^; *SA_es_* is the surface area of the sphere determined by the same volume of the iron ore particle, mm^2^; *V* is the real volume of the iron ore particle, mm^3^.

In the current study, the raw iron ores were obtained from an iron mine in Xuzhou with and iron grade of 67.46%, which are currently used for iron- and steel-making industries in China. The larger chunks of raw iron ore were processed three times by an industrial jaw crusher. Subsequently, the processed particles were reprocessed in a laboratory-scale jaw crusher to obtain the desired product size distribution. Finally, the products were sieved carefully (10 min) by a vibrating screen to obtain the required mono-sized particles. The mono-sized iron ore particles in the range of 2–4 mesh were selected for this study.

**Figure 2:**
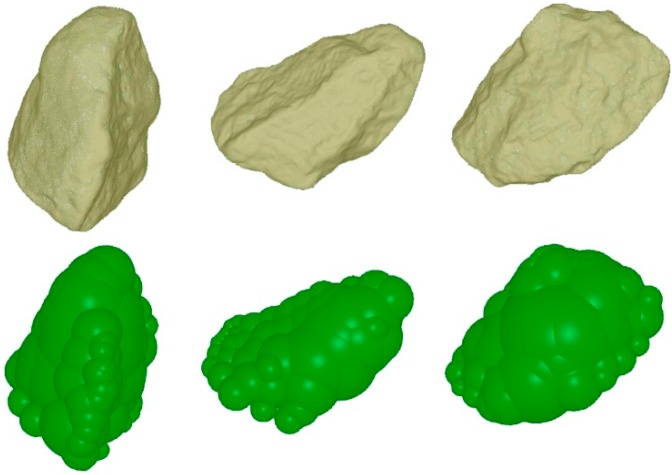
Iron ore particle modelling by the sphere clump method (60 spheres).

In the event that more accurate shape representation was desired, thirty-six particles were selected to generate the 3D digitized and meshed shapes by a high-accuracy 3D scanner (TEXU-BLU, 2M pixel), as shown in Table 1. The 3D scanner used in this study was of 50 × 38 mm in scanning range of a single breadth, 2 s in scanning speed, and ±0.015 mm in scanning accuracy. Subsequently, a quick and accurate sphere clump method was employed in generating the 3D shapes. The elegant software, named Automatic Sphere-clump Generator (ASG), was employed to realize this method for generating a geometrical model that can be used in DEM simulations, as shown in Figure 2. The method is divided into two steps: sphere detection and sphere optimization. The sphere-clump method starts by detecting candidate spheres and then refines the solution using a non-linear least-squares optimization. Since the detection process uses a randomized vertex sampling method to find spheres, different, but equally valid, sphere-clumps can be obtained on consecutive runs. In order to characterize the sphere-clumps quantitatively, two parameters assessing and describing the modelling error, namely volume error and EIT error, were estimated. EIT error shows the percentage average mass distribution error along the principal axes. Volume error shows the percentage error between the mesh volume and clump volume, expressed as:

(11) $$\mathrm{Volum}\;\mathrm{error}=\left|\frac{V_{m}-V_{c}}{V_{m}}\right|\times 100\%$$

where *V_m_* is the mesh volume, and *V_c_* is the clump volume.

### 3.2. Simulation Conditions and Input Parameters

**Figure 3:**
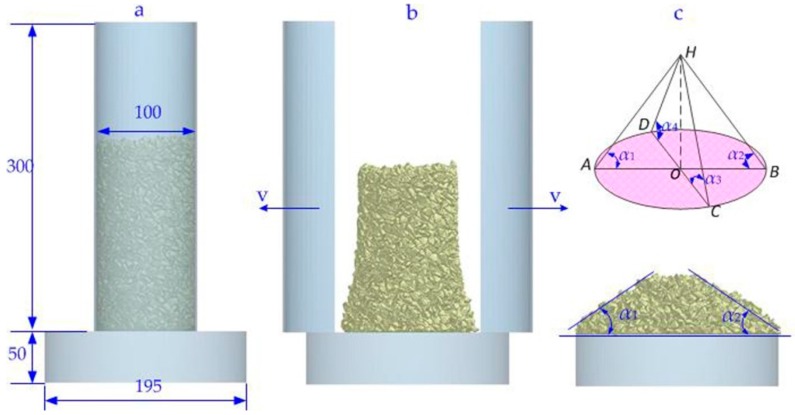
Forming process of the particle pile on the plane. (**a**) Initial state of the simulation; (**b**) being in the particle’s accumulating; (**c**) sand-pile stabilized.

Currently, there are different measurements to determine the angle of repose of particles, including measuring methods, such as the fixed-cone method, fixed-height method, rotating cylinders, and titling method [25,43]. In general, the sand-pile height was measured directly to determine the angle of repose. However, there are some limitations on determining the angle of repose accurately, due to the friction between the particle and the wall. To determine the inter-particle contact parameters purely, a series of DEM simulations in this study were studied on the basis of the swing-arm slump test, originally used by Grima and Wypych [14,44]. Shown in Figure 3a, a base of 50 mm in height and 195 in diameter was fixed horizontally on the ground. A split cylinder of 300 mm in height and 100 mm in diameter was placed on the base. A certain mass of iron ore particles (3 kg) was poured into the cone with a generation rate of 5 kg/s and then the split cylinders were pulled away at a high velocity to avoid the friction between particle and wall. Correspondingly, particles would be collapsed under the natural force of gravitation forming a pile of materials, as shown in Figure 3b, accumulated from the center of the plane and gradually expanding to the boundary. The angle of repose, as shown in Figure 3c, is defined as the angle between the inclined surface between the sand-pile and the plane, and was used to qualitatively analyze the influence of inter-particle contact parameters on particle flow characteristics. Then angle of repose can be approximately calculated and expressed as:

(12) $$\alpha_{AOR}=\frac{45}{\pi }[\sum_{i=1}^{4}\alpha_{i}]$$

where α*_i_* (*i* = 1, 2, 3, 4) is the measured angle in the orthogonal axes with a unit of radians (rad).

Considering the input parameters selected in the EDEM software package (DEM—Solutions Ltd., Edinburgh, UK), the mechanical properties and contact parameters should be determined accurately. The mechanical properties of iron ore particles were obtained using the hydraulic MTS machine, and the mechanical properties of the cone were summarized from the literature. The iron ore particles, in principle, do not have exactly identical mechanical properties because of their distinct microstructure. However, it is extensively recognized that all iron ore particles in this study have the same material properties and contact parameters, due to reductions in the assignment of EDEM input parameters. Researchers presented that the shear modulus was commonly artificially reduced to achieve reasonable simulation times [22]. Hence, the shear modulus used in this study for the iron ore particles has been reduced by a factor of 100 relative to the typical value, and found that the reduced value has no effect on the angle of repose. Currently, the contact parameters for the wall-particle system can be measured accurately, but the inter-particles contact parameters are difficult to determine. To reduce the number of DEM simulations, the contact parameters for the wall-particle system were arbitrarily assumed to be constant, whereas the contact parameters for inter-particles were selected in a range from a low value to a high value (Table 2).

## 4. Results and Discussion

### 4.1. Particle Shape Estimation

**Figure 4:**
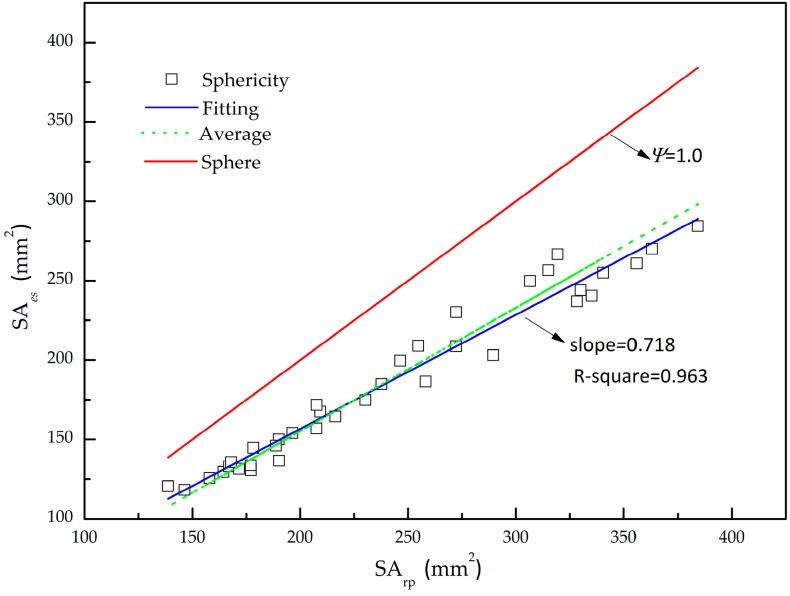
Sphericity of thirty-six iron ore particles.

To determine the mechanical and flow behavior of iron ore particles, thirty-six iron ore particles are selected to study the irregularly shape. The geometrical dimensions of iron ore particles are determined on the basis of the 3D digitized and meshed shapes. Considering the equivalent sphere with the same volume of the real particle, the equivalent surface areas are calculated in Table 1, as shown in Figure 4. The equivalent surface area versus the real surface area is fitted by the method of least squares. The fitting value has a sphericity of 0.718 and a correlation coefficient of 0.963 which is slightly smaller than is case for the average value (0.776), but both are less than the sphericity of a sphere. It is demonstrated that the iron ore particle is highly irregular comparing with the sphere so that the simplifications and assumptions of the irregular particle as simple spherical shape is unrealistic.

**Figure 5:**
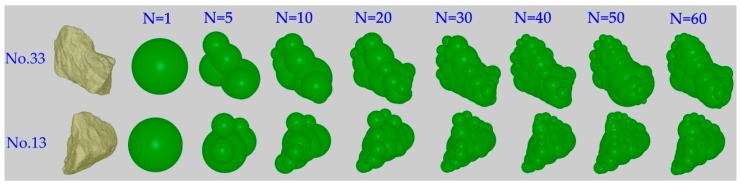
The geometrical model of iron ore particles with various numbers of sphere clumps.

In this study, the no. 13 and 33 are selected to represent the 2 × 4 mesh mono-sized particles, due to the consideration between the average sphericity and the equivalent radius. Other particle sizes are scaled on the basis of the selected particle size, as shown in Table 3. Based on the sphere clump methods, the 3D shapes are generated with a cluster of spheres to represent the irregular model. Studies show that the accuracy of the irregular particle increases with the increasing number of spheres, but at the expense of much greater computational effort. Therefore, considering the accuracy in describing the shape of iron ore particles and computational effort, different numbers of spheres are selected and summarized in Figure 5. It is interesting to see that the accuracy in describing the geometrical shape of iron ore particles increases with increase in the number of spheres. Therefore, considering the accuracy in describing the shape of iron ore particles and computational effort, the appropriate sphere clump numbers should be carefully selected.

**Figure 6:**
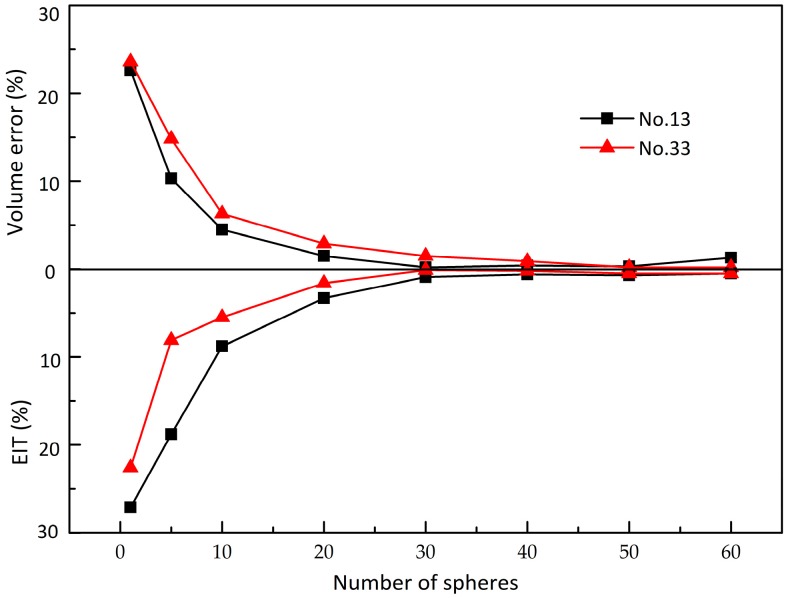
The change in volume error and EIT error with number of sphere clumps.

In this model, the volume error and EIT error describing the details between the 3D model and the geometrical model are obtained, shown in Figure 6. It is also evident from Figure 6 that the change in volume error and EIT error with number of sphere clumps decreases dramatically and then remains approximately unchanged. Once the number of sphere clumps is greater than thirty, the maximum volume error and EIT error are 1.3% and 0.9%, and the minimum volume error and EIT error are 0.2% and 0.1%, respectively. Hence, to achieve computational accuracy without increasing the amount of calculation, the geometrical model used in the work is a cluster of thirty spheres.

**Figure 7:**
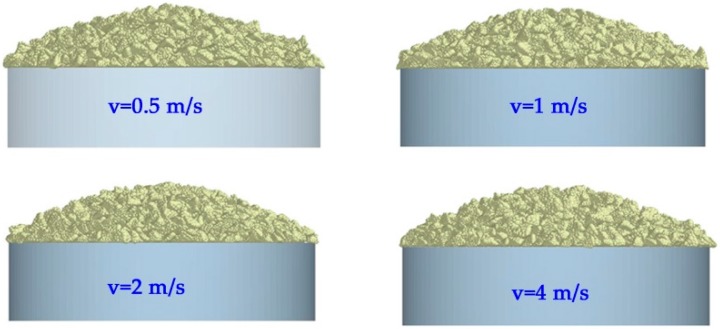
Effect of velocity on angle of speed when *e_pp_* = 0.05, *μ_s-pp_* = 0.15, *μ_r-pp_* = 0.01.

To avoid the friction between the particle and wall, the split cylinder should be pulled away quickly enough. Figure 7 presents the variation of angle of repose as a function of velocity for the given contact parameters. The geometry of the sand-pile and the angle of repose are only a weak variation with the varying of the velocity if the velocity is greater than 0.5 m/s. Hence, in this study, a velocity of 1 m/s is carried out to conduct a series of DEM simulations.

### 4.2. Effect of Inter-Particle Contact Parameters

**Figure 8:**
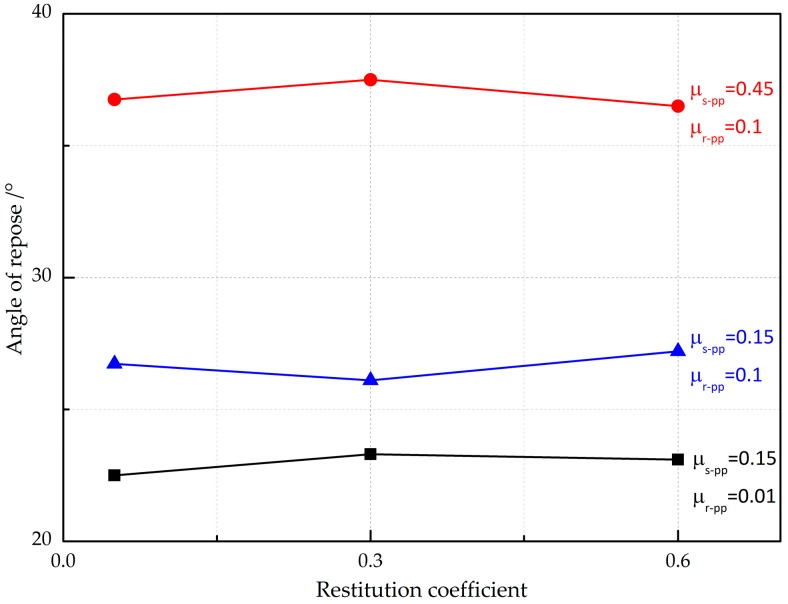
Effect of the restitution coefficient on the angle of repose.

To examine the impact of contact parameters on angle of repose, varying inter-particle contact parameters are systematically changed. The variation of the angle of repose as a function of the restitution coefficient is primarily studied. Figure 8 presents the angle of repose plotted against the restitution coefficient for the given rolling friction coefficient and static friction coefficient. It is clear that the angle of repose varies little with the changing restitution coefficient over the range considered. Additionally, the angle of repose determined by the high friction coefficient is much higher than is case for the low friction coefficient. It is apparent that the angle of repose has strong sensitivity to the rolling/static friction coefficient, but the impacts of the rolling friction coefficient and static friction coefficient on the angle of repose may be a single factor or an interrelated and interdependent effect. As seen in Figure 8, it is clear that the impacts of the rolling friction coefficient and static friction coefficient are interrelated and a combined effect. The restitution coefficient, as a basic property of collision energy, representing the energy dissipation during the collisions, is used to determine the damping coefficient of inter-particle and particle-wall. Thus, varying the damping coefficient slightly leads to the variation of angle of repose. Investigators proposed that the restitution coefficient is not sensitively affected by the collision velocity so that the fixed value of the restitution coefficient is commonly used in the DEM simulations. Therefore, a constant value of the restitution coefficient is used for the rest of the DEM simulations.

**Figure 9:**
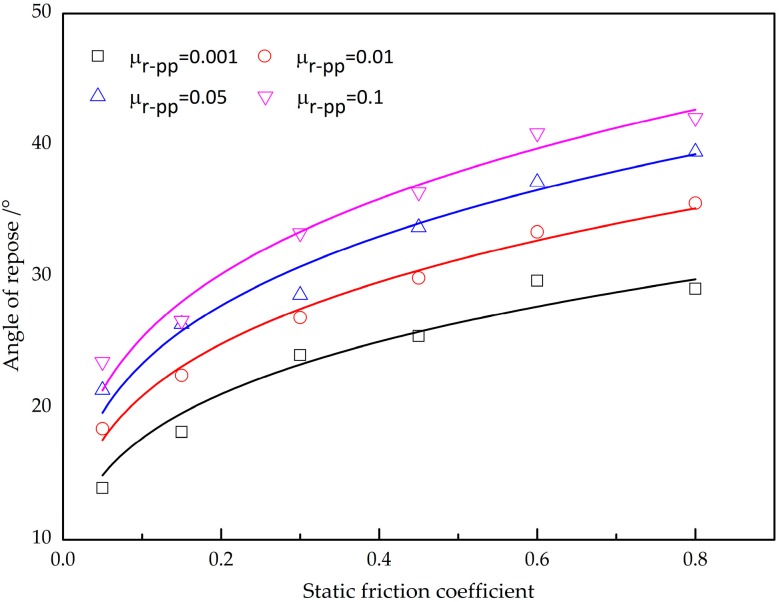
The effect of the static friction coefficient on the angle of repose.

Figure 9 shows the angle of repose as a function of the static friction coefficient under the given restitution coefficient and rolling friction coefficient. The angle of repose is strongly dependent on the static friction coefficient, forming the sand-pile higher, with an increase in the static friction coefficient. It is recognized that the DEM simulation is dependent on the soft contact approach, and the deformation will be generated during the interaction collisions between particles. Larger static friction coefficients can tolerate with a larger elastic deformation in the tangential direction, so it has great potential to form the pile higher for a large static friction coefficient. Additionally, increasing the rolling friction coefficient for the given static friction coefficient increases the angle of repose, and the investigation of the impact of rolling friction coefficient on the angle of repose will be presented in the following section.

**Figure 10:**
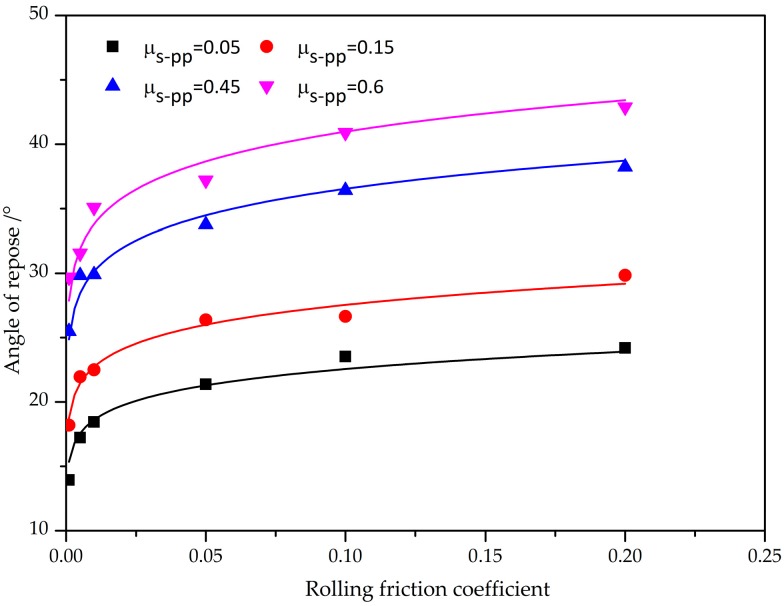
The effect of the rolling friction coefficient on the angle of repose.

Obviously, the rolling friction coefficient and static friction coefficient are both significant in determining the angle of repose, increasing rolling friction coefficient can also obviously increase the angle of repose. Figure 10 shows the angle of repose affected by rolling friction coefficient for the given restitution coefficient and static friction coefficient. Increasing the rolling friction coefficient evidently increases the height of sand-pile, while the bottom width of sand-pile decreases correspondingly. There is a strong dependence with the angle of repose forming higher as the rolling friction coefficient increases. It is mainly because, in the contact model, the rolling friction coefficient actually gives an impactful torque resistance to control the rotational motion of individual particles. A large rolling friction coefficient will provide a large resistance force to control the rotational motion of particles and, therefore, there is more potential to form the sand-pile of iron ore particles higher than is case for the low rolling friction coefficient, largely due to the reduction of a large amount of kinetic energy of particle systems.

### 4.3. Formulation of A Predictive Equation

The above results show that the angle of repose is highly sensitive to the static/rolling friction coefficient, while it appears to be invariant to the restitution coefficient. The simulated angle of repose is fitted to the following relationship using a non-linear equation, giving:

(13) $$\alpha_{AOR}=56.61\cdot {e_{pp}}^{0.01}\mu_{r-pp}^{0.084}\mu_{s-pp}^{0.25}$$

**Figure 11:**
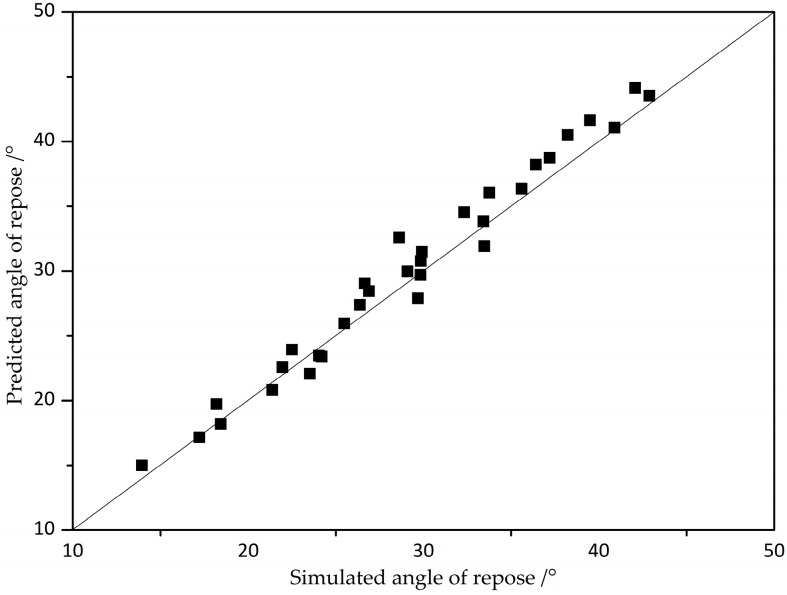
The effect of the simulated angle of repose with the predicted angle of repose.

It is interesting to note that varying the combination of static friction coefficient and rolling friction coefficient can provide an effective prediction to determine the angle of repose of iron ore particles. Based on the non-linear equation, the simulated angle of repose is plotted against the predicted angle of repose, as shown in Figure 11. It is apparent that a very close agreement between the predicted and simulated value is attained. The maximum error is approximately 1.801 degrees, which is almost within the scope of errors.

To investigate further, the indices of each parameter in this equation can also provide the information to determine which inter-particle contact parameter has the greatest impact on the angle of repose. It is clear that the index of the rolling friction coefficient (0.084) and static friction coefficient (0.25) are evidently greater than is the case for the restitution coefficient (0.01). Therefore, it can also be demonstrated that the impact of the restitution coefficient can be neglected whilst the impact of the rolling friction coefficient and static friction coefficient are both significant, compared to the restitution coefficient, in determining the angle of repose, whereas the impact of the static friction coefficient is more profound than that of the rolling friction coefficient. The results are the same to that reported by researchers [45], indicating the applicability of non-linear equations. However, the geometries of iron ore particles are highly irregular, rather than the spherical balls, and the sphere clump method is exclusively employed in determining its geometries. It is essential to recognize that particles in granular processes are usually non-spherical, of varying humidity and particle size in nature. Therefore, the investigations of irregular particles have more universalities than those of spheres. This predictive equation significantly shortened the inter-particle contact parameter calibration time, which can help in the implementation of EDEM simulations.

## 5. Conclusions

The results presented above contribute to a better understanding of the impact of inter-particle contact parameters of mono-sized iron ore particles, such as the restitution coefficient, rolling friction coefficient, and static friction coefficient, on the angle of repose on the basis of discrete element method (DEM). In particular, it was shown that the iron ore particle was highly irregular with a sphericity of 0.718, and then the particles were modelled by a sphere clump method. The angle of repose was found to show a weak function of the restitution coefficient, whereas it had strong sensitivity to the static friction coefficient and rolling friction coefficient with a combined effect. Therefore, the fixed value of the restitution coefficient is commonly used in the DEM simulations. Furthermore, to address which parameter has the greatest impact on the angle of repose, a predictive equation was established to determine the inter-particle contact parameters. A very close agreement between the predicted and simulated angle of repose was attained. It was clear that the indices of the rolling friction coefficient (0.084) and static friction coefficient (0.25) were evidently greater than is the case for the restitution coefficient (0.01). Notably, the static friction coefficient plays a primary role on the angle of repose, followed by a less, but still profound, role for the rolling friction coefficient. The predictive equation can be very helpful to determine the inter-particle contact parameters of iron ore particles and can also significantly shorten the calibration time.

## Figures and Tables

**Table 1:** The real physical properties of thirty-six particles using a high-accuracy 3D scanner.

No	*x* (mm)	*y* (mm)	*z* (mm)	*SA_rp_* (mm^2^)	*V* (mm^3^)	*SA_es_* (mm^2^)	Ψ
1	12.00	10.13	9.04	306.59	371.26	249.76	0.815
2	17.28	10.96	9.32	384.21	450.97	284.34	0.740
3	16.33	8.81	10.56	362.99	417.46	270.08	0.744
4	17.08	7.66	10.56	356.01	396.36	260.90	0.733
5	11.99	11.10	9.00	315.12	386.53	256.56	0.814
6	8.90	7.80	6.57	177.12	140.36	130.59	0.737
7	9.06	10.32	8.40	207.58	184.78	156.86	0.756
8	7.27	6.81	7.92	157.98	132.59	125.72	0.796
9	9.87	6.98	6.58	190.06	172.74	149.97	0.789
10	7.68	8.26	7.16	164.40	138.55	129.47	0.787
11	10.87	9.99	5.76	216.31	198.35	164.45	0.760
12	8.66	4.68	9.18	196.37	179.84	154.05	0.785
13	10.06	7.24	7.52	188.76	165.99	146.04	0.77
14	6.90	6.68	5.60	138.72	124.48	120.54	0.87
15	9.78	5.50	8.02	177.04	145.33	133.65	0.75
16	7.25	13.93	10.87	330.06	358.60	244.05	0.74
17	7.73	15.17	9.13	335.25	350.82	240.511	0.72
18	14.14	7.09	8.76	272.36	283.56	208.69	0.77
19	13.98	8.13	8.80	319.42	409.57	266.66	0.83
20	10.51	8.15	8.50	246.43	265.3	199.65	0.81
21	14.23	8.60	9.19	340.58	383.07	255.03	0.749
22	14.40	10.72	5.92	289.56	272.20	203.08	0.701
23	11.70	13.77	6.36	328.38	343.20	237.02	0.722
24	10.23	8.57	10.07	272.33	328.38	230.14	0.845
25	9.44	11.33	5.86	254.76	284.11	208.96	0.820
26	14.01	7.77	7.67	258.21	239.39	186.41	0.722
27	10.33	6.63	10.22	237.68	236.24	184.78	0.777
28	12.54	6.90	7.06	230.27	217.36	174.80	0.759
29	10.26	7.30	9.09	209.29	203.88	167.49	0.800
30	9.04	8.21	7.40	207.62	211.68	171.73	0.827
31	10.84	6.59	6.823	190.17	150.06	136.54	0.718
32	9.02	7.13	6.232	167.01	144.34	133.05	0.797
33	7.10	8.25	7.374	171.66	141.85	131.51	0.766
34	6.94	8.27	8.773	178.29	163.69	144.69	0.812
35	8.66	6.82	7.359	167.92	148.69	135.71	0.808
36	8.11	7.29	6.48	146.37	120.78	118.14	0.807

**Table 2:** Input parameters for EDEM simulations.

Material Parameters	Symbols	Value
Particle density (kg m^−3^)	*ρ_p_*	3886
Particle shear modulus (Gpa)	*G_p_*	2.587
Particle Poisson’s ratio	*ν_p_*	0.283
Wall density (kg m^−3^)	*ρ_w_*	1200
Wall shear modulus (Gpa)	*G_w_*	1.05
Wall Poisson’s ratio	*ν_w_*	0.41
Particle-wall restitution coefficient	*e_pw_*	0.5
Particle-wall static friction coefficient	*μ_s-pw_*	0.6
Particle-wall rolling friction coefficient	*μ_r-pw_*	0.05
Particle-particle restitution coefficient	*e_pp_*	0–0.6
Particle-particle static friction coefficient	*μ_s-pp_*	0–0.8
Particle-particle rolling friction coefficient	*μ_r-pp_*	0–0.2

**Table 3:** Percent of the particle size used in the simulations.

Volume Intervals (mm^3^)	Percent
100–200	44.44%
200–300	25%
300–400	22.22%
400–500	8.33%
